# The Sale of Poisons in Bombay

**Published:** 1866-02

**Authors:** 


The Sale of Poisons in Bombay.—A new Bill has been introduced into the Legislative Council of Bombay, to regulate and restrict the sale of poisons in the Presidency, in assimilation to the English Acts.—*London Lancet.*

